# Conformations of voltage-sensing domain III differentially define Na_V_ channel closed- and open-state inactivation

**DOI:** 10.1085/jgp.202112891

**Published:** 2021-08-04

**Authors:** Paweorn Angsutararux, Po Wei Kang, Wandi Zhu, Jonathan R. Silva

**Affiliations:** 1 Department of Biomedical Engineering, Washington University in St. Louis, St. Louis, MO; 2 Department of Medicine, Brigham and Women’s Hospital, Boston, MA

## Abstract

Voltage-gated Na^+^ (Na_V_) channels underlie the initiation and propagation of action potentials (APs). Rapid inactivation after Na_V_ channel opening, known as open-state inactivation, plays a critical role in limiting the AP duration. However, Na_V_ channel inactivation can also occur before opening, namely closed-state inactivation, to tune the cellular excitability. The voltage-sensing domain (VSD) within repeat IV (VSD-IV) of the pseudotetrameric Na_V_ channel α-subunit is known to be a critical regulator of Na_V_ channel inactivation. Yet, the two processes of open- and closed-state inactivation predominate at different voltage ranges and feature distinct kinetics. How inactivation occurs over these different ranges to give rise to the complexity of Na_V_ channel dynamics is unclear. Past functional studies and recent cryo-electron microscopy structures, however, reveal significant inactivation regulation from other Na_V_ channel components. In this Hypothesis paper, we propose that the VSD of Na_V_ repeat III (VSD-III), together with VSD-IV, orchestrates the inactivation-state occupancy of Na_V_ channels by modulating the affinity of the intracellular binding site of the IFMT motif on the III-IV linker. We review and outline substantial evidence that VSD-III activates in two distinct steps, with the intermediate and fully activated conformation regulating closed- and open-state inactivation state occupancy by altering the formation and affinity of the IFMT crevice. A role of VSD-III in determining inactivation-state occupancy and recovery from inactivation suggests a regulatory mechanism for the state-dependent block by small-molecule anti-arrhythmic and anesthetic therapies.

## Teaser

Angsutararux et al. propose that the voltage-sensing domains of repeats III and IV of Na_V_ channels regulate state-dependent inactivation by modulating the conformation and affinity of the IFMT motif found in the linker between the III and IV repeats.

## Introduction

Voltage-gated Na^+^ (Na_V_) channels initiate excitation in neurons and myocytes, enabling rapid conduction over large distances that is decoupled from intracellular Ca^2+^ signaling (Hille, 2001). During excitation, Na_V_ channel opening releases a large inward Na^+^ current (I_Na_) that is followed by rapid inactivation, which occurs within milliseconds and makes most channels nonconductive. This inactivation from the open state is required to allow outward repolarizing currents to bring the cell membrane back to the resting potential. However, inactivation recovery is not instantaneous at hyperpolarized membrane potentials, nor is it limited to depolarized potentials. For example, in cardiac myocytes, Na_V_ channels remain inactivated for 10s to 100s of milliseconds following action potential repolarization, rendering the myocyte refractory to excitation for a brief period and preventing reentrant arrhythmia (Zipes et al., 2017). In addition, neuronal memory of previous excitation can be conferred by inactivation of Na_V_ channels that reduces the subsequent firing rate (Marom, 1998; Toib et al., 1998). At modest depolarized membrane potentials (less than −30 mV), Na_V_ channels can also become inactivated before the activation gate opening but require longer depolarizations (Aldrich et al., 1983; Bean, 1981). This closed-state inactivation results in fewer available channels to initiate excitation. Thus, the regulation of Na_V_ channel inactivation by time and membrane potential is essential for the functioning of excitable cells, allowing neurons and myocytes to appropriately respond to changes in membrane potential that span multiple time domains (Silva, 2014).

**Figure 1.**
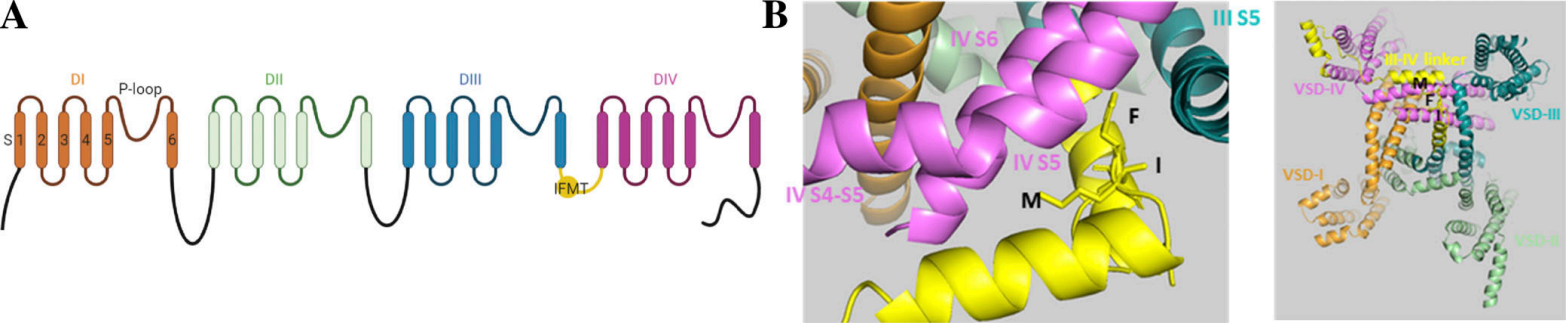
**The Na_V_ channel inactivation mechanism.(A)** A schematic representation of mammalian Na_V_ channel shows four homologous repeats (I–IV) and the IFMT motif on the III–IV linker (yellow). **(B)** The structure of rNa_V_1.5 (PDB accession no. 6UZ3) shows the IFMT motif enclosed by repeat III S5 and repeat IV S4–S5 linker and S5 and S6 segments, causing an allosteric block during inactivation.

Mammalian Na_V_ channels are formed by a single protein with four homologous repeats (I–IV; Fig. 1 A), each constituting six membrane-spanning segments (S1–S6). Within each repeat contains a voltage-sensing domain (VSD) that consists of segments S1–S4. The Na^+^-selective pore is formed jointly by the S5 and S6 segments (Yu and Catterall, 2003), with the region between S5 and S6 constituting the P-loop responsible for Na^+^ ion selectivity. Mammalian Na_V_ channels additionally feature intracellular linkers and a unique C-terminal domain (CTD), which is known to bind various accessory subunits, including calmodulin and fibroblast growth factor homologous factors (Abriel, 2010). Given the complex time and voltage dependence of Na_V_ channel inactivation, it must be connected to multiple different conformational states, whose occupancy is determined by the positions of the VSDs, the state of the channel pore, and the position of the CTD (Ulbricht, 2005).

Indeed, several reports have connected the activation of the VSD-IV to the onset of fast inactivation after channel opening (Capes et al., 2013; Goldschen-Ohm et al., 2013). Mutations in VSD-IV also suggest a role in closed-state inactivation (Kambouris et al., 2000; Chahine et al., 1994; Groome et al., 2011). Charge neutralization mutations within VSD-IV cause a large hyperpolarizing shift in voltage-dependent channel availability and a large fraction of inactivated channels at voltages where the channels are closed (Capes et al., 2013; Brake et al., 2021 *Preprint*). Early experiments demonstrated that the addition of intracellular pronase removed Na_V_ channel inactivation and revealed the participation of intracellular components (Armstrong et al., 1973; Armstrong, 1981; Salgado et al., 1985). The model of Na_V_ channel inactivation was then suggested to resemble the “ball-and-chain” model of K^+^ channel N-type inactivation, where a ball that is attached to the inner part of the channel causes inactivation by occluding the pore (Armstrong and Bezanilla, 1977; Hoshi et al., 1990). Subsequent experiments that disrupted the intracellular linker between repeats III and IV produced functional channels but lacked inactivation (Stühmer et al., 1989; Vassilev et al., 1988; Vassilev et al., 1989), further confining the sites of the inactivation gate to the III-IV linker. Finally, site-specific mutagenesis identified a hydrophobic cluster of amino acids, Ile-Phe-Met-Thr (IFMT), as an essential component for the inactivation mechanism (Hartmann et al., 1994), and a “hinged-lid” model was proposed (Eaholtz et al., 1994; Kellenberger et al., 1997; Rohl et al., 1999; West et al., 1992). In this model, the loop between two hinged points serves as a rigid lid that folds over the channel pore, with the IFMT motif acting as a hydrophobic latch to stabilize the inactivated conformation. Additional mutagenesis and the discovery of inherited proarrhythmic mutations within cardiac Na_V_ channels further suggested that the binding site or the “receptor” for the IFMT motif involves repeats III and IV S4–S5 linkers and IV S6 segment (Smith and Goldin, 1997; McPhee et al., 1995; McPhee et al., 1998). Recent structures of eukaryotic Na_V_ channels, however, reveal that the IFMT motif resembles more of a wedge that squeezes into the crevice formed by repeat III S5 and repeat IV S4–S5 linker and S5 and S6 segments (Yan et al., 2017; Pan et al., 2018, 2019; Shen et al., 2019; Jiang et al., 2020; Li et al., 2021) and allosterically blocks the Na_V_ channel conduction pore (Fig. 1 B).

Apart from the inactivation gate, the VSD-III, the CTD, and the state of the channel pore have also been implicated in Na_V_ channel inactivation (Cha et al., 1999; Deschênes et al., 2001; Hsu et al., 2017; Mantegazza et al., 2001; Motoike et al., 2004; Pitt and Lee, 2016, Mangold et al., 2017). Based on new structural data in combination with numerous previous functional studies, we hypothesize that the activation of the VSD-III, in addition to the VSD-IV, facilitates different inactivated states by modulating the binding affinity of IFMT crevice. In this Hypothesis paper, we will first outline the models of each inactivated state. Then, we will present the structural and electrophysiological evidence that supports our hypothesis and leads to the proposed model. Finally, we will discuss the model implications on the modulation of Na_V_ channel function by accessory subunits and therapeutic drugs.

## The models of open- and closed-state Na_V_ channel inactivation

**Figure 2.**
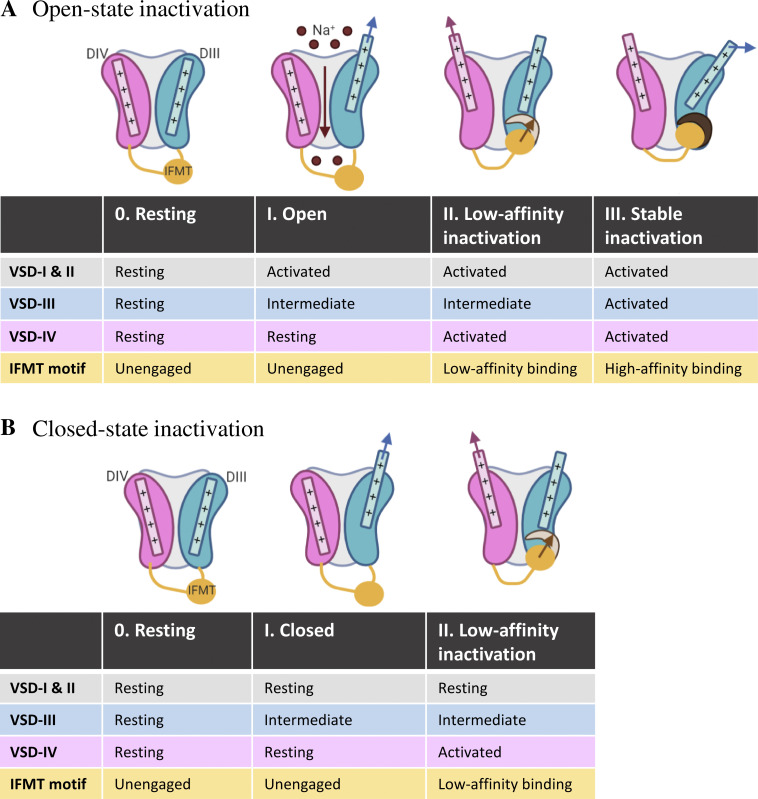
**The models of open- and closed-state inactivation based on two VSD-III depolarized conformations.(A)** During open-state inactivation, strong membrane depolarization leads to the activation of repeat I and II VSDs and the intermediate activation of VSD-III, resulting in the opening of the activation gate and the conduction of Na^+^ ions (I). Activation of the VSD-IV exposes the low-affinity binding site for the IFMT motif (II). Further translation of the VSD-III into its fully activated conformation establishes the stable inactivated configuration with high-affinity IFMT motif binding (III). **(B)** For closed-state inactivation at hyperpolarized membrane potential, VSD-III activates while repeats I and II VSDs are at rest, resulting in closed activation gate and no I_Na_ (I). Subsequent VSD-IV activation forms the low-affinity binding site for the IFMT motif (II). Since VSD-III occupies primarily in its intermediate conformation, the stable inactivated configuration is not established.

We propose a VSD-III and VSD-IV-centric inactivation model (created at https://biorender.com; Fig. 2), which incorporates structural motifs throughout the channel. In this model, the VSD-III can activate in two distinct conformations over different voltage ranges as detected by fluorescent tracking of voltage sensor conformational change upon membrane depolarization (Chanda and Bezanilla, 2002; Zhu et al., 2017) and various supportive electrophysiological evidence (Varga et al., 2015; Hsu et al., 2017). The intermediate and fully activated conformations of VSD-III then unmask distinct crevices with low and high affinity for the IFMT motif, as suggested by eukaryotic Na_V_ channel structures (Yan et al., 2017; Jiang et al., 2020), that leads to unique kinetics of closed- versus open-state inactivation. Different inactivated states are therefore connected to distinct activated conformations of VSD-III.

During the process of open-state inactivation (Fig. 2 A), the VSDs of repeats I and II rapidly move outward into their activated position upon membrane depolarization. At the same time, VSD-III adopts its intermediate conformation, and the activation gate opens (step I), allowing the channel to pass current. Shortly thereafter, VSD-IV activates and a partial crevice for the IFMT motif is formed. The III–IV linker may also be released from CTD interaction upon VSD-IV activation, as suggested by the structure of the α-scorpion toxin AaH2-bound hNa_V_1.7 VSD-IV–Na_V_PaS hybrid, where the intracellular end of resting IV S4 (K7 and R8) interacts with the CTD, causing the sequestration of the III–IV linker (Clairfeuille et al., 2019). The IFMT motif can move into the hydrophobic cleft formed by repeat IV S4–S5 linker and S6 segment and establish low-affinity inactivation binding (step II). Concurrently, further translation of VSD-III into its fully activated conformation repositions the III S4–S5 linker to increasingly interact with the helix along III–IV linker, providing additional support for the stable inactivated conformation (step III). The binding of the IFMT motif leads to a rearrangement around S6 helices and an allosteric block of the conduction pathway. During fast inactivation, the VSDs of repeats I and II are free to deactivate upon membrane hyperpolarization. VSD-III and VSD-IV, however, are immobilized by the IFMT motif. Importantly, the deactivation of VSD-III and VSD-IV determines inactivation recovery time course, with the slowness of VSD-III deactivation playing a rate-limiting role (Hsu et al., 2017).

For closed-state inactivation, at membrane potentials less than −30 mV (Fig. 2 B), VSD-III activates to its intermediate conformation while VSD-I and VSD-II are at rest (step I). When VSD-IV moves up to its activated conformation, the crevice for IFMT motif is partially exposed and low-affinity IFMT binding is established (step II). Because of the hyperpolarized potential, VSD-III primarily occupies only its intermediate conformation, incapable of providing supportive interactions around the inactivation gate, and thus the channel is unable to adopt a stable inactivated configuration. The inactivated state with low-affinity IFMT motif binding results in the slow kinetics of closed-state inactivation. As the VSD-IV activates at more positive potentials than the VSD-III (Varga et al., 2015), the rate limiting of closed-state inactivation is reflected by the overlap in voltage range at which VSD-IV activates and the steady-state inactivation occurs (Capes et al., 2013).

In the following section, we will review supporting evidence that underlies the key aspects and implications of our proposed hypothesis.

## Differential Na_V_ channel–inactivated states are determined by the IFMT binding affinity that is modulated by the conformations of repeats III and IV VSDs

The closed and open Na_V_ channel inactivation states differ not only in the voltage range over which they are occupied but also prominently in their kinetics, where inactivation proceeds at a much faster rate from the open-channel conformation (Aldrich et al., 1983; Goldman, 1995). As shown by structural and functional studies, inactivation is due to the binding of the IFMT motif to a crevice (Fig. 1 B). If the final step in both closed- and open-state inactivation is identical, then how can these two inactivation processes feature distinct kinetics? One possible explanation is that the conformation of IFMT binding site is dynamic and its binding affinity can be tuned. The crevice is formed by the S4–S5 linker of repeat IV, S5 of repeat III, and the S5 and S6 segments of repeat IV with additional interactions from repeat III S4–S5 linker aiding in the stabilization of the IFMT motif binding, as indicated by many recent structures (Yan et al., 2017; Pan et al., 2018; Shen et al., 2019; Pan et al., 2019; Jiang et al., 2020) and past mutagenesis studies (Kellenberger et al., 1997; McPhee et al., 1995, 1998; Smith and Goldin, 1997).

In the resting state model of rat Na_V_1.5 (rNa_V_1.5), fitted after the resting bacterial Na_V_Ab structure (Wisedchaisri et al., 2019), the IFMT crevice is blocked by the intracellular end of repeat III S4 and the S4–S5 linkers of repeats III and IV (Jiang et al., 2020). The outward translation of repeat III and IV VSDs is therefore a prerequisite for rendering the inactivation crevice available. The movement of III– or IV–S4 segments will pull on their corresponding S4–S5 linkers and position them accordingly. In other words, the extent of VSD activation (i.e., the number of gating charges that moves from an internal to an external side) directly affects the placement of the S4–S5 linker, which in repeats III and IV form the integral component of the IFMT crevice. Thus, different combinations of the VSD-III and VSD-IV depolarized conformations may give rise to different binding affinities of the IFMT crevice, resulting in the distinct inactivation kinetics observed over different membrane potentials. We expect that there are also differences in the contribution from VSD-III and VSD-IV in regulating the IFMT crevice between different Na_V_ channel homologues (Chanda and Bezanilla, 2002; Varga et al., 2015; Brake et al., 2021 *Preprint*).

Facilitation of inactivation by the depolarized VSD-IV conformation has been convincingly shown via multiple charge neutralization and toxin studies (Capes et al., 2013; Chen et al., 1996; Kontis et al., 1997; Kühn and Greeff, 1999; Clairfeuille et al., 2019). Further studies have revealed that VSD-III may also play a key role in modulating IFMT binding. During fast inactivation, a fraction of the gating charge was found to be immobilized by the inactivation gate (Armstrong and Bezanilla, 1975; Bezanilla and Armstrong, 1974; Bezanilla et al., 1982). These gating charges were identified as components of the repeat III and IV VSDs, suggesting their interaction with the III–IV linker (Armstrong and Bezanilla, 1977; Cha et al., 1999). Specific disruption of the inactivation particle through an IFM/ICM mutation did not eliminate the immobilization of VSD-IV, yet entirely abolished VSD-III immobilization (Sheets and Hanck, 2005), emphasizing a functional linkage between the IFMT motif and the VSD-III depolarized conformation.

The role of VSD-III in regulating Na_V_ channel closed- and open-state inactivation was additionally highlighted through various protocols. Particularly, biasing VSD-III to the depolarized conformation by tethering extracellular MTSEA-biotin on repeat III S4 through an R3C mutation dramatically reduced Na_V_ channel availability via enhanced closed-state inactivation and its slope factor but showed minimal effects on the peak I–V relationship (Sheets and Hanck, 2007). The R1128C (R4C on repeat III S4 segment) mutation in rat skeletal muscle sodium channel (rNa_V_1.4) promoted closed-state inactivation, leading to reduced steady-state availability (Groome et al., 2011, 2014a). Similar mutations (R4H on repeat III S4 segment) in human skeletal muscle Na_V_ channel (hNa_V_1.4 R1135H) and human cardiac sodium channel (hNa_V_1.5 R1309H) showed enhanced entry into inactivated states with prolonged recovery from inactivation (Groome et al., 2014b; Wang et al., 2016). When the three outermost gating charges of VSD-III were neutralized, the fraction of inactivated channels at subthreshold potentials (−60 and −50 mV), increased and the delay to the onset of open-state inactivation after a conditioning pulse decreased, but to a lesser extent than the neutralization of VSD-IV (Capes et al., 2013). The charge neutralization in VSD-III of Na_V_1.5 caused the similar significant shift in steady-state inactivation curve toward hyperpolarizing potentials, which is an indicative of increased closed-state inactivation, as observed when the VSD-IV charges were neutralized (Brake et al., 2021 *Preprint*). All these results illustrate that the modulation of the repeat III S4 either through a chemical manipulation or a reduction in the number of VSD-III gating charges that facilitate VSD-III activation enhance closed-state inactivation and reduce channel availability, emphasizing the significant role of VSD-III activated conformations in regulating the states of Na_V_ channel inactivation.

Interestingly, open-state and closed-state inactivation feature distinct kinetics: open-state inactivation kinetics show little change in rate over the voltages at which it occurs (Aldrich et al., 1983), whereas the time constant of closed-state inactivation is highly voltage dependent (Goldman, 1995; Sheets and Hanck, 1995). An inactivation gate peptide (KIFMK) that mimics the IFMT motif and restores the fast inactivation in defective mutant Na_V_ channels illustrates weak voltage dependence in terms of inactivation time constants at depolarized potential of more than −30 mV (Eaholtz et al., 1994; Peter et al., 1999). This observation, together with the absence of gating current component with a time course that tracks inactivation, suggests that inactivation after channel opening has no intrinsic voltage dependence (Armstrong, 2006). We hypothesize that the voltage dependence of closed-state inactivation arises from movements of the VSDs of repeats III and IV exposing the crevice for IFMT binding, while during open-state inactivation, rapid and complete VSD-III and VSD-IV activation fully reveals the crevice and inactivation kinetics are determined by voltage-independent III-IV linker diffusion. Previously, closed-state inactivation was postulated to occur when the repeats III and IV VSDs move to depolarized conformations, while the VSDs of repeats I and II remain at rest (Armstrong, 2006). We further propose that the position of the VSD-III is crucial in determining the conformation of the IFMT binding site and that inactivation state occupancy is derived from the multiple steps traversed by the VSD-III as it activates. In the next section, we will examine studies that imply different VSD-III activated conformations.

## Unique VSD-III activated conformation determines the differential binding of the IFMT motif

Recent structures of eukaryotic Na_V_ channels featured components of Na_V_ channel inactivation machinery that were missing from prior structures of bacterial Na_V_ channels. The first eukaryotic Na_V_ channel structure from the American cockroach, Na_V_PaS, captured closed pore domain with all four VSDs in an “activated” conformation (Shen et al., 2017), defined by two positively charges within the S4 segment having crossed the hydrophobic constriction site (HCS), a cluster of hydrophobic residues that prevents ion leakage in voltage sensor (Yang et al., 1996; Starace and Bezanilla, 2004; Tao et al., 2010; Catterall, 2014). The III–IV linker, although similar in length to other eukaryotic Na_V_ channels, lacks the key hydrophobic motif required for inactivation and is shown to interact with the CTD. Subsequently, the electric eel Na_V_1.4 (eeNa_V_1.4), human Na_V_1.2 (hNa_V_1.2), hNa_V_1.4, hNa_V_1.7, and rat Na_V_1.5 (rNa_V_1.5) structures were resolved. These structures are all highly similar, with high resolution of key elements that are critical for inactivation mechanism. In these structures, the III–IV linker contains the inactivation motif (LFM for eeNa_V_1.4) binding to the crevice formed by repeat III S5, repeat IV S4–S5 linker, and S5 and S6 segments (Fig. 1 B), likely representing the inactivated state structure (Yan et al., 2017; Pan et al., 2018; Shen et al., 2019; Pan et al., 2019; Jiang et al., 2020). All four VSDs are also captured in the activated positions, albeit some with different conformations in the Na_V_PaS structure. The CTD is not well resolved and is thus omitted.

**Figure 3.**
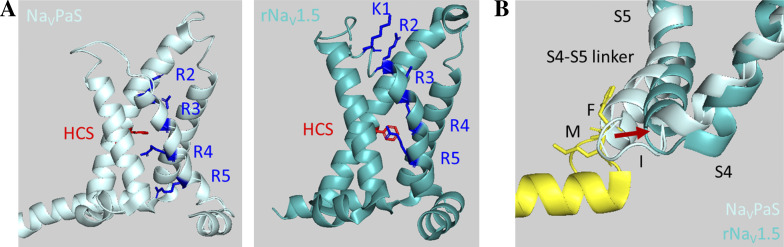
**A comparison between Na_V_PaS and rNa_V_1.5 structures. (A)** Structures of eukaryotic Na_V_ channel reveal two distinct conformations of the depolarized VSD-III, varying in the number of gating charges (K and R) across the HCS. In Na_V_PaS (left, light blue; PDB accession no. 5X0M), the activated VSD-III transfers two positively charged residues from an internal to an external side. The other VSD-III activated conformation from rNa_V_1.5 (right, cyan; PDB accession no. 6UZ3) captures the total of four gating charges transfer. **(B)** An overlay of two Na_V_ channel structures shows that the VSD-III fully activated conformation (cyan) is needed to facilitate the binding of the IFMT motif.

Comparison between Na_V_PaS and other eukaryotic Na_V_ channels reveals VSD-III can activate in two steps. First, the positions of the VSD-III differ in the extent of their depolarized conformation or activation. Even though Na_V_PaS channel is nonfunctional and may not represent a relevant physiological state, Na_V_PaS contains highly conserved transmembrane segments that are similar to and superimpose well with other eukaryotic Na_V_ channels. The number of arginines on repeat III S4 segment is also comparable among two groups. Differences observed may provide an opportunity for dissecting Na_V_ channel mechanism. In Na_V_PaS, two gating charges on repeat III S4 are transferred across the HCS, while in other structures, a total of four charges on III S4 segment are displaced from the intracellular to the extracellular side (Fig. 3 A). These structural variations reveal that the VSD-III might adopt two distinct activated conformations correlating to different numbers of charges across the HCS. The trajectory of the VSD-III activation resembles a sigmoidal curve, with an intracellular tip moving laterally away from the pore and the helix rotating upward and bending downward at the extracellular end (Yan et al., 2017).

**Figure 4.**
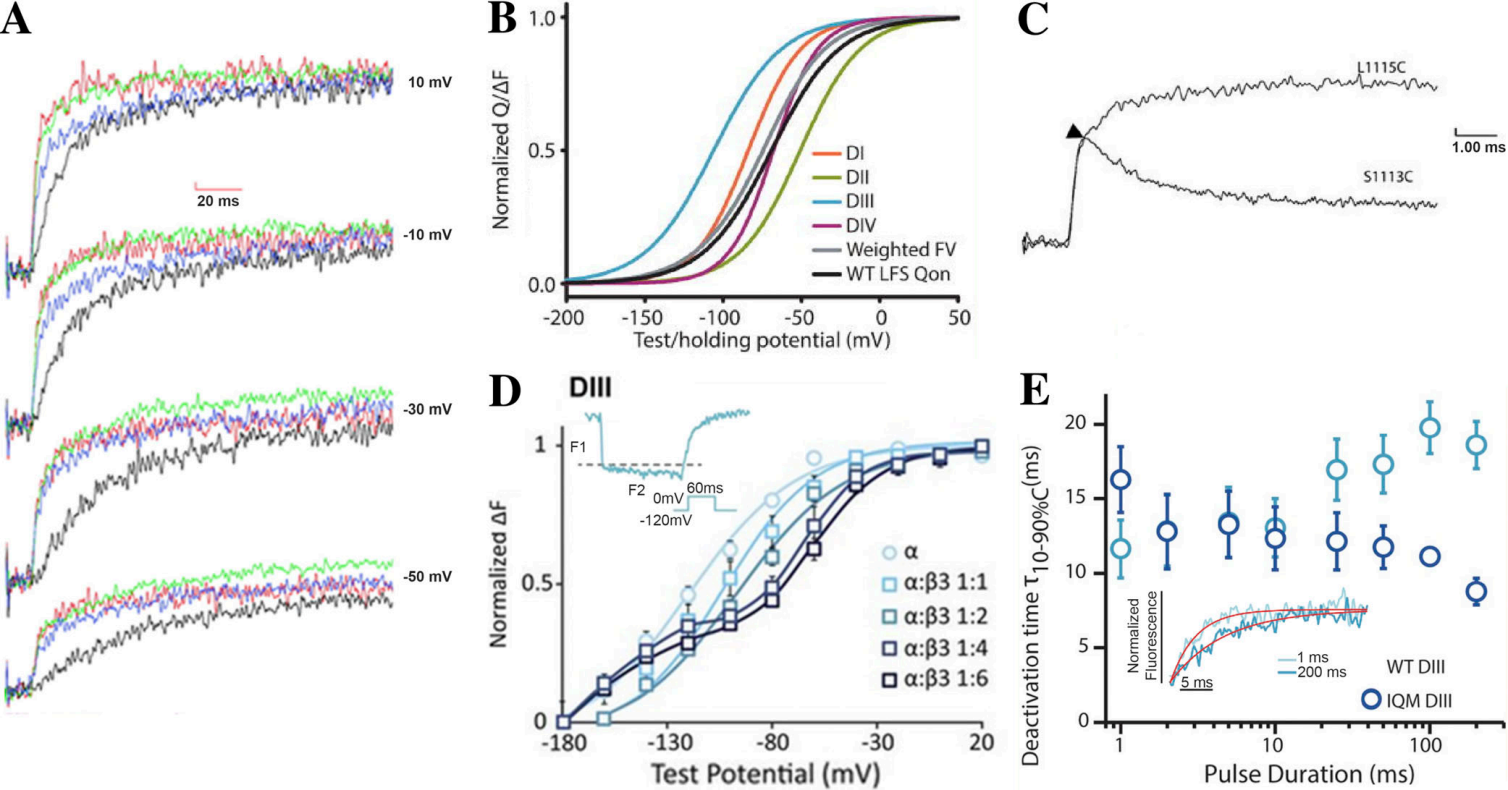
**Experimental data supporting a two-step VSD-III activation model. (A)** VCF recordings of rNa_V_1.4 show simultaneous initiation of repeats I–III VSD activation (blue, red, and green) but a delay in VSD-IV activation (black), which is reduced with increasing voltages. Adapted from Chanda and Bezanilla (2002). **(B)** Fluorescence–voltage (FV) curves of hNa_V_1.5 VCF constructs show that VSD-III activates at hyperpolarized potential, earlier than other repeat VSDs. Adapted from Varga et al. (2015). LFS, large fluorescence signal. **(C)** VCF recording of S1113C in rNa_V_1.4 elicits two stages of VSD-III movement, with an initial increase followed by a decrease in fluorescence emission. Adapted from Chanda and Bezanilla (2002). **(D)** Coexpression of hNa_V_1.5 and β3-subunit at high ratio (1:4 and 1:6) yields two distinct components of VSD-III activation kinetics, as detected by fluorescence emission (inset) and fluorescence–voltage curves. Adapted from Zhu et al. (2017). **(E)** A correlation between VSD-III deactivation time constants and the depolarization duration in WT hNa_V_1.5 suggests the multiple steps of VSD-III activation, which is dependent on the IFMT motif as illustrated by the loss of correlation in IQM mutation. Adapted from Hsu et al. (2017). Error bars represent the standard error of the mean from the sample size of 3–6 measurements.

Two-step VSD-III activation is further supported by functional experiments that tracked Na_V_ channel VSD movement optically via the voltage-clamp fluorometry (VCF) technique (Zhu et al., 2016; Rudokas et al., 2014; Chanda and Bezanilla, 2002; Mannuzzu et al., 1996). The VCF protocol was developed to observe the conformational dynamics of individual S4 segments and can reveal structurally correlated kinetic information (Cowgill and Chanda, 2019). By attaching a fluorophore to the extracellular S3–S4 linker site through a cysteine mutation, the movement of the proximal S4 segment can be detected upon the changes in fluorescence emission caused by differences in the surrounding environment (Cha et al., 1999b; Glauner et al., 1999; Bezanilla, 2000; Chanda and Bezanilla, 2002). Simultaneous recordings of fluorescence emission and ionic current identified the concurrent activation of repeat I–III VSDs during Na_V_ channel activation, with a slight delay of VSD-IV activation lagging the rise of the I_Na_ (Fig. 4 A). The VSD-III is most sensitive to hyperpolarized potentials, having the half-maximal voltage of the fluorescence–voltage curve at the most negative potential among all VSDs (Fig. 4 B; Varga et al., 2015). The VSD-III is already activated at highly negative potentials where closed-state inactivation occurs.

Two stages of VSD-III activation were demonstrated in the VCF recordings of rNa_V_1.4 VSD-III with a fluorophore attached to S1113C, showing an initial increase in the fluorescence emission followed by a reduction (Fig. 4 C; Chanda and Bezanilla, 2002). Similarly, in another independent recording of hNa_V_1.5 in the presence of high β3-subunit expressions, the fluorescence emission tracking VSD-III activation at M1296C showed two components with distinct kinetics (Fig. 4 D; Zhu et al., 2017). The curves also displayed two elements of VSD-III activation, implying that VSD-III activates with different kinetics over different voltage ranges. Recently, the VCF recordings of neonatal form Na_V_1.5 (Na_V_1.5e) reported the biphasic relationship of VSD-III fluorescence–voltage curve reaching its maximum at −50 mV and declining with more depolarized potentials (Brake et al., 2021 *Preprint*). Finally, the time constant of VSD-III deactivation, unlike other VSDs, varied with depolarization duration, hinting at the multiple-step VSD-III activation mechanism (Fig. 4 E; Hsu et al., 2017). If the VSD activation progressed to only a single conformation, then prolonged depolarization would not affect the time it takes to return to its resting position. Instead, when the S4 segment travels across two distinct states, longer depolarizing pulses bias the transition to the second step and result in longer deactivation times. Altogether, extensive structural and functional evidence strongly supports two-step VSD-III activation.

The two conformations of VSD-III activation lead to an interesting consequence that each activation step may dictate a different conformation of the IFMT crevice. Because of the highly similar structures in the transmembrane core and the same length of III-IV linker across eukaryotic Na_V_ channels, the relative positions of repeat III S4 and its ensuing S4–S5 linker can provide relevant, useful insights. In the Na_V_PaS structure, the position of the III S4 segment orients the S4–S5 linker so that it interferes with IFMT binding and likely obstructs the formation of the IFMT crevice (Fig. 3 B). The short section on the III–IV linker before the IFMT motif is also restricted from moving near the membrane, further constraining the range over which the IFMT motif can travel. In contrast, further translation of the III S4 in other eukaryotic structures pulls the S4–S5 linker up and away from the pore such that a crevice is exposed and direct interactions with the IFMT motif can be established (Fig. 3 B; Jiang et al., 2020). Consecutive helices on the III–IV linker provide additional interactions with the S4–S5 linkers of repeats III and IV that further stabilize inactivated state. Specifically, charged residues near the IFMT motif allosterically interact with repeats III and IV VSDs (Groome et al., 2003; Groome et al., 2007).

Electrostatic interactions from the III–IV linker cause the immobilization of repeats III and IV VSDs during prolonged depolarization (Armstrong and Bezanilla, 1977; Kuo and Bean, 1994; Cha et al., 1999). The recovery from inactivation is thus affected by the kinetics of VSD-III and VSD-IV deactivation. When the IFMT motif was mutated to IQMT, fast inactivation was greatly impaired, and the deactivation of repeats III and IV VSDs was significantly accelerated (Hsu et al., 2017). The time constant of VSD-III deactivation that normally increases with depolarizing pulse duration no longer shows such correlation in the IQMT mutant channel (Fig. 4 E), suggesting that IFMT binding specifically stabilizes the second VSD-III activated state (Hsu et al., 2017). The same study also showed that inactivation recovery after >200 ms depolarization is defined exclusively by the deactivation of VSD-III (Hsu et al., 2017). This result coincides with the second step VSD-III activation, transferring the highest number of gating charges across the HCS and hence likely returning last to the resting position. Other arrhythmogenic mutations along the IFMT binding sites on S4–S5 linkers of III and IV (N1325S, A1330, and N1659A) also showed enhanced recovery from inactivation, accompanying the decreased time constant of VSD-III deactivation (Hsu et al., 2017). Together, the differential states of VSD-III activation are likely to provide the basis for distinct Na_V_ channel inactivation kinetics at hyperpolarized and depolarized potentials.

## Physiological and pharmacological modulation of repeat III VSD activation affects Na_V_ channel inactivation

Multiple factors involved in the regulation of Na_V_ channel inactivation, such as the β-subunits, drugs and toxins, were shown to modulate VSD-III activation. Resolved structures of hNa_V_1.2, hNa_V_1.4, and hNa_V_1.7 with coexpressed β1-subunit identified its docking site near the VSD-III with interactions between the Ig domain of β1-subunit and repeat IV L6 (extracellular linker between P-loop and S6) and repeat I L5 (extracellular linker between S5 and P-loop) loops (Pan et al., 2018; Shen et al., 2019; Pan et al., 2019). Such interaction sites imply β-subunit modulation of VSDs III and IV kinetics. Careful study of the mechanism of noncovalent β-subunits (β1 and β3) demonstrated a unique impact on the initiation of repeat III and IV VSDs activation, which results in distinct modulatory effects on the hNa_V_1.5 (Zhu et al., 2017). Consistent with the resolved structures, coexpression of β3-subunit shifts VSD-III and VSD-IV activation, leading to altered steady-state inactivation, activation, and fast inactivation kinetics. Switching the charge of a β3-subunit transmembrane glutamic acid (E176K) altered VSD-III activation of hNa_V_1.5 and affected the channel recovery from inactivation (Salvage et al., 2019). To the contrary, the β1-subunit regulates Na_V_ channel inactivation solely through the modulation of the VSD-IV, resulting in a shift in steady-state inactivation, while having no impact on I_Na_ activation or fast inactivation kinetics (Zhu et al., 2017). This unique interaction between cardiac Na_V_ channel and β1-subunit is further supported by rNa_V_1.5 and hNa_V_1.5 structures that failed to resolve the coexpressed β1-subunit (Jiang et al., 2020; Li et al., 2021). The different regulating mechanisms between β1 and β3 subunits on hNa_V_1.5, therefore, emphasize the significance of the VSD-III conformation in determining the state and kinetics of Na_V_ channel inactivation.

The action of the local anesthetic (LA) lidocaine has also been intimately linked to VSD-III function. LAs are normally used for local and regional anesthesia and the treatment of excitatory pathologies, including epilepsy and cardiac arrhythmia. Their significant therapeutic effect is achieved through use-dependent block, an increase in channel blocking over repetitive activation. This effect is due to the higher-affinity binding when the channel is in the open and inactivated states (Bean, 1981), resulting in a cumulative increase of inactivated channels over successive pulses of depolarization. The modulated receptor hypothesis postulates that the accessibility to the high-affinity binding site depends on the state of the channel, which in turn involves voltage-dependent VSD activation (Hille, 2001). Lidocaine stabilizes VSD-III in its activated state in rNa_V_1.4 channel coexpressed with β1-subunit (Muroi and Chanda, 2009). The prepositioning of both repeats III and IV VSDs in the outward position (through MTSEA-biotin modified R3C III and R2C IV) enhances lidocaine block significantly in hNa_V_1.5 α-subunit alone, and when the inactivation gate is disrupted via IFM/ICM mutation, the stabilization of individual III or IV S4 increases lidocaine affinity similar to when both VSDs are stabilized, suggesting an equally significant contribution of VSD-IV as VSD-III to lidocaine block (Sheets and Hanck, 2003; Sheets and Hanck, 2007). The discrepancy that is observed between two studies by Muroi et al. and Sheets et al. could be attributed to the intrinsic properties of different Na_V_ channel isoforms as well as the presence of β1-subunit. We found that lidocaine induces hyperpolarizing shifts in VSD-III and VSD-IV activations, but β1-subunit coexpression subdues the VSD-IV modulation (Zhu et al., 2021). The inactivation gate, though not necessary for drug binding, drastically reduces drug binding affinity when it is removed (Bennett et al., 1995; Sheets and Hanck, 2007). How the inactivation gate contributes to the kinetics of lidocaine block is still not fully understood (Fozzard et al., 2011).

It is, nevertheless, interesting to note the parallel molecular determinants between lidocaine and the IFMT motif binding affinity. Both are dependent upon the positions of VSD-III and VSD-IV. Even though lidocaine is found to bind in the inner pore of Na_V_ channel through interaction with phenylalanine and tyrosine on the repeat IV S6 segment (Ragsdale et al., 1994; Yarov-Yarovoy et al., 2002; F1759 and Y1766 in Na_V_1.5) and directly blocks Na^+^ conductance, binding of the IFMT motif on the rim of the activation gate might also shape the local interaction around the lidocaine-binding site. For one, the IFMT binding stabilizes the outward positions of VSD-III and VSD-IV, which are integral to the drug receptor formation. Thus, the VSD translocation of repeats III and IV could have twofold implications, directly through the VSD-pore coupling and indirectly through the binding of the IFMT motif. According to our hypothesis, the preactivation of VSD-III by lidocaine would favor the high-affinity binding of the IFMT motif and facilitate the inactivation, which in turn enables the high drug affinity–binding mode. A chemical accessibility study of the inactivation gate position during lidocaine block in rNa_V_1.4 F1304C (IFMT motif) by MTSET modification showed that lidocaine favors the inactivation gate closure, hypothetically by promoting the transition along the activation pathway (Vedantham and Cannon, 1999).

Finally, a study of a lidocaine derivative, mexiletine, also shows that the level of VSD-III activation strongly affects drug efficacy (Zhu et al., 2019; Moreno et al., 2019). Mexiletine preferentially blocks late I_Na_, which is enhanced in many cardiac pathologies, including long-QT type 3 syndrome. Investigation into varied mexiletine sensitivity of different long-QT type 3 mutations identified a correlation to the fraction of activated VSD-III. These results resonate with the findings from the lidocaine studies on the significance of VSD-III position on a determination of LA like drug affinity. Taken together, these studies of Na_V_ channel accessory subunits and its blockers emphasize the essential role of VSD-III activation in the regulation of Na_V_ channel inactivation kinetics.

## Future perspective

There is often a singular focus on individual channel domains in the regulation of the Na_V_ channel inactivation, consistent with the m^3^h gating model of Hodgkin and Huxley, where a single “h” gate mediates inactivation. However, a myriad of studies has proved the process to be more elaborate and complicated than the simplified representation of this model, and intricate inactivation is not unique to Na_V_ channels. Homotetrameric potassium channels and prokaryotic Na_V_ channels display complexity in the coupling of inactivation to channel activation (Hoshi et al., 1990; Yang et al., 2018). Toxin and pharmacological studies suggest the possibility of a drug-induced alteration in Na_V_ channel conducting conformation (Baumgarten et al., 1991) or a unique drug-bound inactivated state (Finol-Urdaneta et al., 2019a; Finol-Urdaneta et al., 2019b; Ong et al., 2000). Distinct channel isoforms also tend to respond to drug and toxin differently, such as the differential sensitivity to tetrodotoxin or a unique external lidocaine-binding site in the cardiac Na_V_ channel (Baumgarten et al., 1991). More importantly, variabilities in the voltage-dependent activation and inactivation curves among different Na_V_ isoforms suggest their distinctive propensity to closed-state inactivation that likely stems from the unique sequence of VSDs activation (Brake et al., 2021 *Preprint*) determined by their rates of activation and the midpoint of activation curve (Capes et al., 2013). Because of the heterogeneity and complexity of the channel kinetics, combined results from multiple methods should be interpreted with careful consideration.

Through multiple Na_V_ channel structures and electrophysiological studies combined with the fluorescent labeling technique, we argue here that VSD-III, together with VSD-IV, governs the binding affinity for the inactivation gate and thus affects the different inactivated states. Still missing, however, is the discrete structure of state-dependent conformations such as those of the resting-state and closed-state inactivation, where some VSDs are not activated. One possible means to overcome this obstacle is the use of toxin, small-molecule modulators, or biochemical methods to force certain states of the channel (Clairfeuille et al., 2019; Wisedchaisri et al., 2019; Moreno et al., 2019). Linking channel structure to channel functional states is also challenging but is achievable via dynamic tools such as FRET. FRET is a distance-dependent energy transfer between two fluorophores, namely the excited donor and the emitting acceptor (Sekar and Periasamy, 2003), that can be attached to any protein or peptide. This method allows for the measurement of molecular proximity between two different parts within angstrom distances and thus is often used to test molecular interactions. When combined with patch clamp, FRET measurement could be used to determine the interactions between the channel’s different domains at various functional states. Integrating such knowledge to the available structures would provide the means to derive a state-dependent structure and a dynamic change in its conformation. Recent work by Kubota et al. (2017) used a lanthanide-based resonance energy transfer, which allows distance measurement, to map the voltage sensor positions of rNa_V_1.4 during the resting and inactivated states. To gain better insight into Na_V_ channel inactivation, the ability to label the intracellular linkers or region is critical. Conventional fluorescent proteins and genetically encoded tags produce large stable fluorescent signals but are disruptive to the protein structure due to their bulkiness (Toseland, 2013). To overcome such limitations, genetic code expansion through site-specific mutation of fluorescent unnatural amino acids such as ANAP (Shandell et al., 2019; Kalstrup and Blunck, 2017) could serve as potential alternatives. Using these techniques to track activation gating and intracellular inactivation particle binding over different membrane potentials would allow differentiation of the states of inactivation, namely closed and open, and ultimately dissection of the regulatory components required for the physiological function of Na_V_ channels.
